# Physico-chemical properties and toxicological effects on plant and algal models of carbon nanosheets from a nettle fibre clone

**DOI:** 10.1038/s41598-021-86426-5

**Published:** 2021-03-25

**Authors:** Syed Shaheen Shah, Mohammed Ameen Ahmed Qasem, Roberto Berni, Cecilia Del Casino, Giampiero Cai, Servane Contal, Irshad Ahmad, Khawar Sohail Siddiqui, Edoardo Gatti, Stefano Predieri, Jean-Francois Hausman, Sébastien Cambier, Gea Guerriero, Md.Abdul Aziz

**Affiliations:** 1 Center of Research Excellence in Nanotechnology, King Fahd University of Petroleum and Minerals, Dhahran, 31261 Saudi Arabia; 2 Department of Life Sciences, University of Siena, via P.A. Mattioli 4, 53100 Siena, Italy; 3 TERRA Teaching and Research Center, Gembloux Agro-Bio Tech, University of Liège, 5030 Gembloux, Belgium; 4 Environmental Research and Innovation (ERIN) Department, Luxembourg Institute of Science and Technology (LIST), 5, avenue des Hauts-Fourneaux, Esch-sur-Alzette, Luxembourg; 5 Life Sciences Department, King Fahd University of Petroleum and Minerals (KFUPM), Dhahran, 31261 Saudi Arabia; 6 School of Biotechnology and Biomolecular Sciences (BABS), The University of New South Wales, Sydney, NSW 2052 Australia; 7 Institute of Bioeconomy (IBE), National Research Council, Via P. Gobetti, 101-I, I-40129, Bologna, Italy; 8 Environmental Research and Innovation (ERIN) Department, Luxembourg Institute of Science and Technology, 5, rue Bommel, Z.A.E. Robert Steichen, 4940 Hautcharage, Luxembourg

**Keywords:** Plant sciences, Materials science

## Abstract

Carbon nanosheets are two-dimensional nanostructured materials that have applications as energy storage devices, electrochemical sensors, sample supports, filtration membranes, thanks to their high porosity and surface area. Here, for the first time, carbon nanosheets have been prepared from the stems and leaves of a nettle fibre clone, by using a cheap and straight-forward procedure that can be easily scaled up. The nanomaterial shows interesting physical parameters, namely interconnectivity of pores, graphitization, surface area and pore width. These characteristics are similar to those described for the nanomaterials obtained from other fibre crops. However, the advantage of nettle over other plants is its fast growth and easy propagation of homogeneous material using stem cuttings. This last aspect guarantees homogeneity of the starting raw material, a feature that is sought-after to get a nanomaterial with homogeneous and reproducible properties. To evaluate the potential toxic effects if released in the environment, an assessment of the impact on plant reproduction performance and microalgal growth has been carried out by using tobacco pollen cells and the green microalga *Pseudokirchneriella subcapitata*. No inhibitory effects on pollen germination are recorded, while algal growth inhibition is observed at higher concentrations of leaf carbon nanosheets with lower graphitization degree.

## Introduction

Carbon (C) nanomaterials obtained from renewable resources are attracting much interest in light of their sustainability. Several papers have indeed reported the manufacture of porous C nanomaterials (like hierarchical porous C nanosheets-CNS) from several types of plant biomass, such as waste coffee grounds^1^, leaves of tal palm^2^, hemp fibres^3^, cornstalk^4^, pomelo peels^5^, bamboo shoots^6^, ginkgo leaves^7^ and even flower petals presenting a layered structure^8^. Plant biomass is cheap and renewable, two important factors that contribute to comply with the requirements of a sustainable economy. Additionally, the anisotropy and porosity of plant components, such as wood, is an interesting feature to consider when preparing CNS, since ion transfer is promoted and electrode material tortuosity alleviated^9^.

The particular cell wall composition and structure of plant tissues confers hierarchical structure to CNS, as shown for the hemp bast fibre-derived CNS^3^: the crystalline cellulosic cell walls confer partial graphitic order to the CNS after KOH activation at high temperature.

The final characteristics of the C nanomaterials deriving from plant biomass depend on the homogeneity of the biological material. Plants growing in the field under changing environmental conditions are subjected to several (a)biotic cues which can impact the biomass yield, as well as the phytochemical content^10–13^. Therefore, cultivating clones (instead of varieties) under controlled conditions enables to collect biological material with homogeneous properties.

Herbaceous plants are an attractive source of biomass, as they produce lignocellulosics in a short time, as compared to woody species. Fibre crops are such an example of fast-growing herbaceous plants and, among them, underutilized species, such as stinging nettle (*Urtica dioica* L.) contribute to diversify the current fibre crop market and produce molecules with interesting bioactivities^14–16^.

Recent papers have reported the cell wall composition and gene expression pattern in different stem internodes of a fibre clone of nettle, clone 13^17,18^, grown under controlled conditions. The presence of mature bast fibres with a thick gelatinous cell wall was observed in older stem internodes^18^.

In this study, for the first time, highly porous activated CNS have been synthesized from the leaves and stems of a stinging nettle (*U. dioica*) fibre clone via pyrolysis at 650 °C and by using NaHCO_3_ as an activating agent. The detailed structure and morphology of the resulting nettle-derived CNS have been here characterized by X-ray diffraction (XRD) and field emission scanning electron microscopy (FESEM), whereas their elemental compositions monitored by energy dispersive X-ray spectroscopy (EDS). The choice of a clone bred for increased fibre yield is motivated by the willingness to use a homogeneous starting material in terms of morphology and composition.

Large-scale production of nanomaterials could, however, lead to a significant release and accumulation of these compounds in the environment, as experienced with other synthetic materials. Due to their nano- and/or micrometric size, such materials could be dispersed into the air and, once aero-dispersed, they could be transported over long distances and come into contact with both terrestrial and aquatic organisms. This raises important concerns about possible negative impacts caused by their release. At present, different (positive and negative) effects of nanocomposites on terrestrial plants and algae have been reported due to different experimental conditions and species tested^19–22^. To evaluate the impact of nettle CNS if released in the environment, the effects on plant cells and microalgae have been studied by using tobacco (*Nicotiana tabacum* L.) pollen as a model of terrestrial plant cells and *Pseudokirchneriella subcapitata* as a model of aquatic organism.

## Results and discussion

### Surface morphology and porosity of CNS

Plant biomass is chiefly composed of cellulose. This biopolymer is composed for approximately 44% of C; however, the C content can increase to around 80% with pyrolysis at high temperatures and reach 95% using various activating agents^23,24^. The preparation of activated C involves two steps: (i) the pyrolysis of biomass in an inert atmosphere and (ii) the activation of carbonized C with activating agents. The former step produces C from raw materials and the latter is used to enlarge the diameters of fine pores and develop new pores after the carbonization step^25,26^. The morphology, porous nature and specific surface area of C nanomaterials derived from plant biomass mainly depend on the activating agents, e.g. ZnCl_2_, KOH, NaOH, H_3_PO_4_ and carbonates^2,27,28^. Among them, sodium bicarbonate (NaHCO_3_) is a well-known activating agent used to generate pores in C nanomaterials and increase their specific surface area^2,28^. Pyrolysis at 650 °C and NaHCO_3_ were used to prepare CNS from nettle biomass. The surface morphology and porous nature of the nettle-derived CNS were investigated through FESEM measurements.

**Figure 1:**
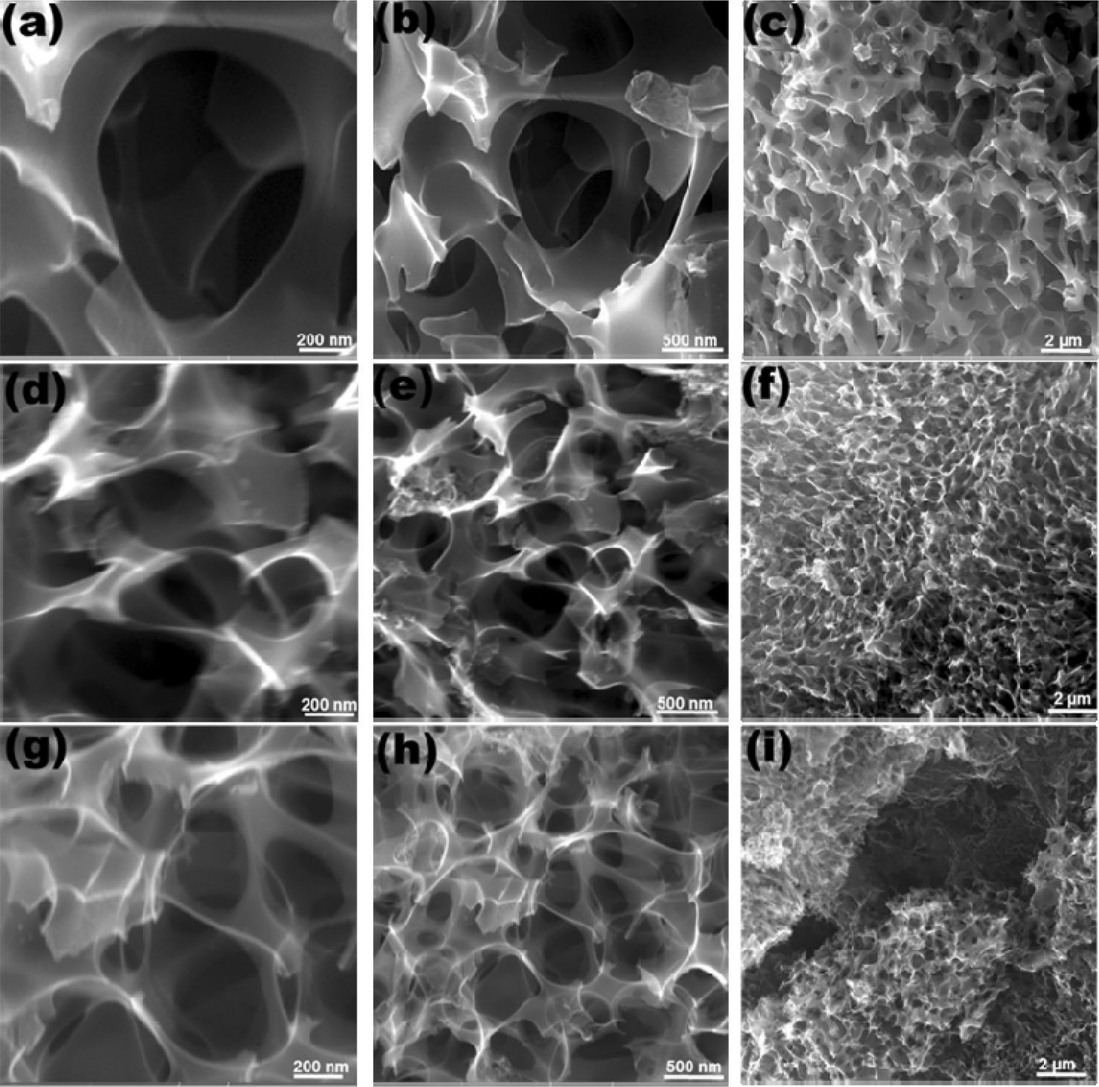
FESEM images at different magnification of CNS prepared at 650 °C from nettle stems (**a**–**c**) and leaves (**d**–**i**) using NaHCO_3_ as an activating agent. The ratio of nettle powder and NaHCO_3_ is 1:1 (**a**–**f**) and 1:2 (**g**–**i**).

The FESEM micrographs of the CNS obtained with 1:1 and 1:2 nettle powder: NaHCO_3_ ratio show an interconnected nanosheet-like structure containing a network of pores (Fig. 1). Figure 1(panels a–c) shows the FESEM images at different magnification of CNS1:1 prepared from nettle stems, while Fig. 1(d–f and g–i) those relative to the nettle leaf-derived CNS1:1 and CNS1:2, respectively. The chemical activation of CNS using NaHCO_3_ treatment at a high temperature forms pores in the C skeleton, thus resulting in the formation of interconnected highly porous CNS. The ImageJ software (ImageJ bundled with 64-bit Java 1.8.0_172, https://imagej.nih.gov/ij/download.html) was used to measure the thickness of the prepared CNS: the average thicknesses were 36, 32 and 27 nm for the nettle stem-derived CNS1:1 (Fig. 1b) and leaf-derived CNS1:1 (Fig. 1e) and CNS1:2 (Fig. 1h), respectively. It was observed that changing the ratio of activating agent and nettle powder did not alter the morphology of the CNS. The above results clearly indicate that NaHCO_3_ activation can increase the active surface area of the prepared C by producing pores. The porous structure of the C is useful for several applications, such as supercapacitor, electrochemical sensing^29^ (fast diffusion of the electrolyte^2^) and to increase the surface area for biological applications^30^.

### Elemental analysis of CNS

**Figure 2:**
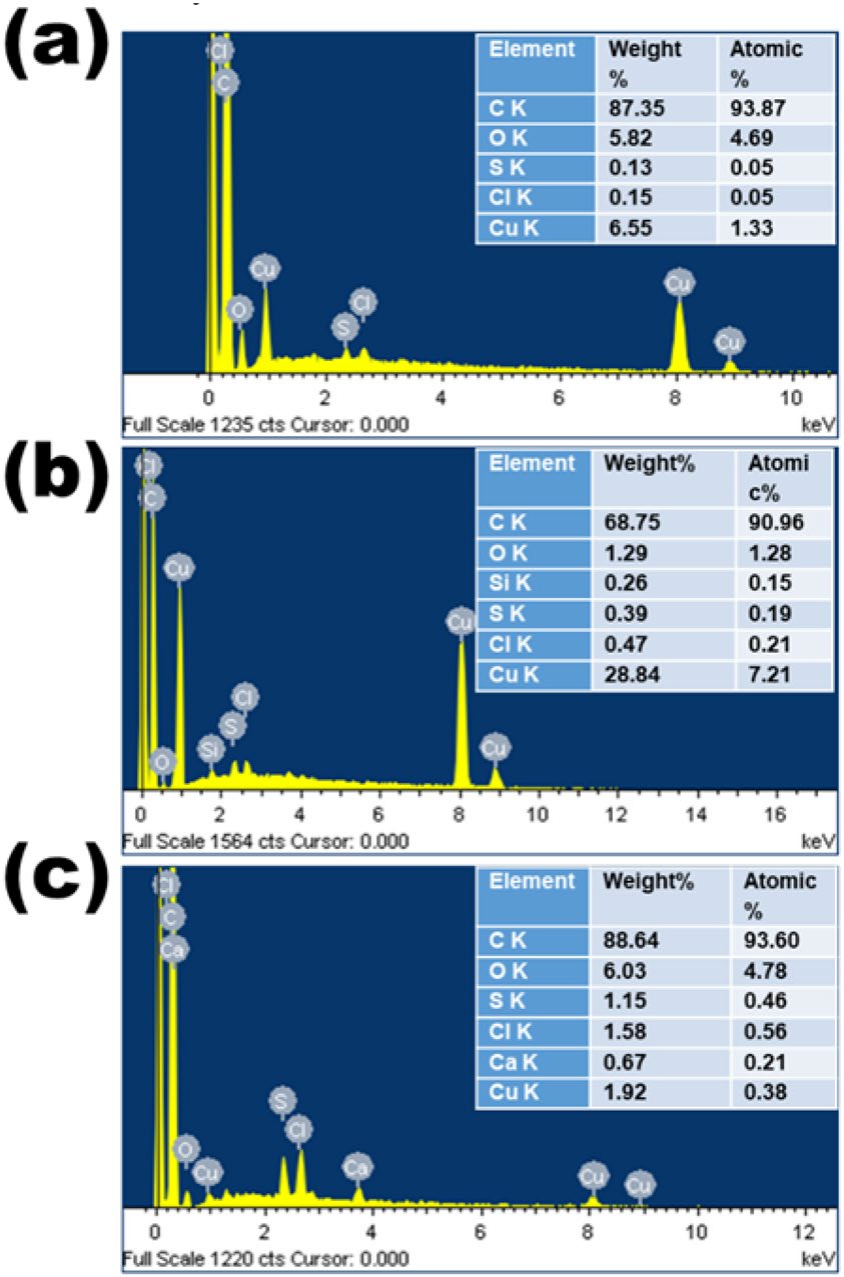
EDS spectra of CNS prepared at 650 °C from nettle stems (**a**) and leaves (**b**,**c**) using NaHCO_3_ as an activating agent. The ratio of nettle powder and NaHCO_3_ is 1:1 (**a**,**b**) and 1:2 (**c**).

EDS was used to detect the presence of elements in the prepared C samples. Both qualitative and quantitative EDS results are shown in Fig. 2. These results indicate the presence of very high C content (> 90% atomic weight) as a major element and low O content (between 1.29 and 6.03% atomic weight) as a minor element in all the prepared CNS. Traces of Ca, Si and Cl are also present, which generally occur in plant tissues^31^ and, consequently, in plant-derived C nanomaterials^32^. Additionally, nettle is known as a silicifier, with its trichomes typically containing silica at the tip^33^.

Some trace amounts of Cu and S can also be seen in all the prepared samples: the presence of Cu is due to the substrate, because of the drop drying of CNS on the surface of Cu tape used as conductive support. S is a fake peak around 2.3 eV and derives from the combination of O and Si; more specifically, it is a sum of O Kα and Si Kα peaks.

**Figure 3:**
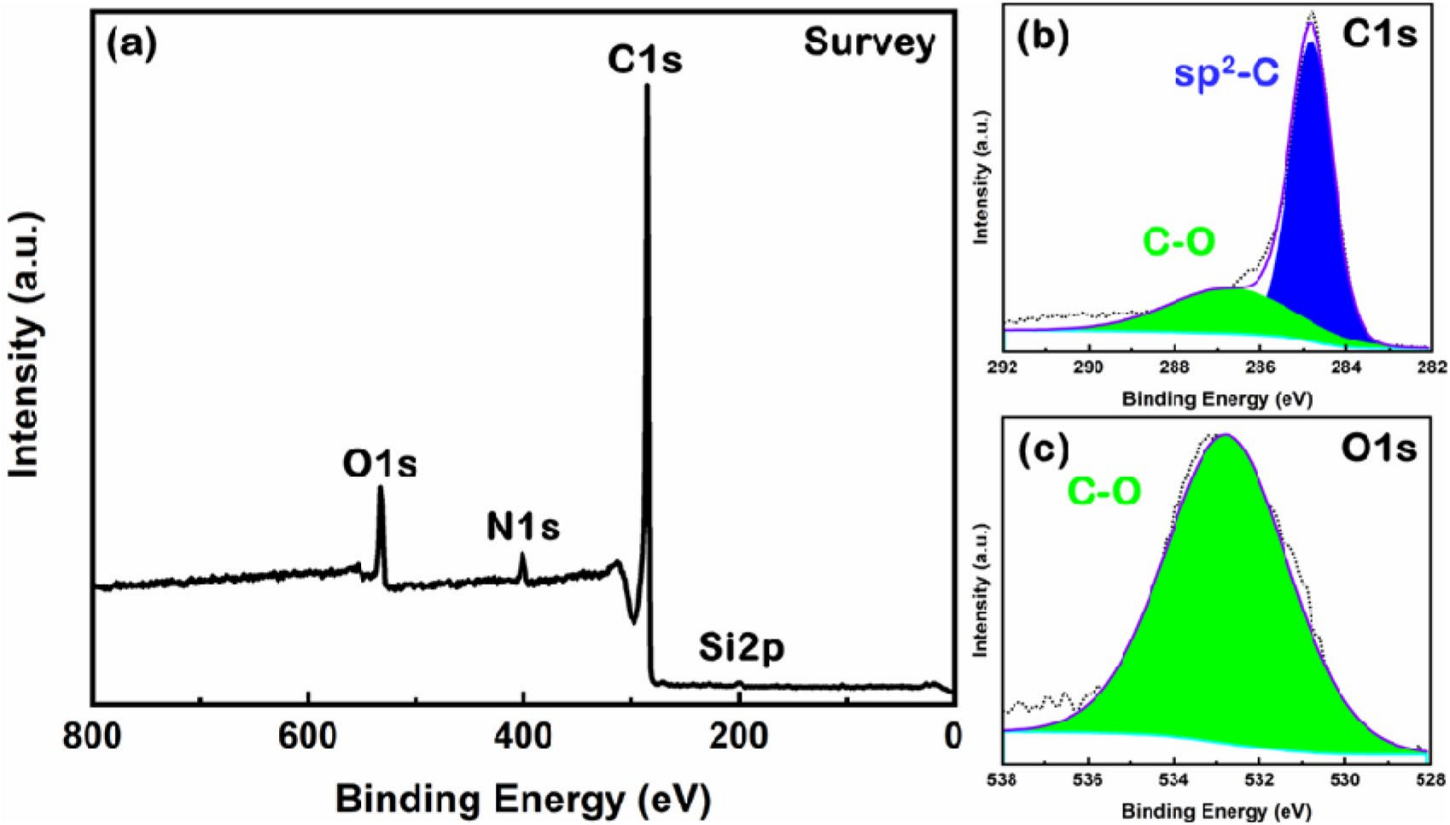
XPS analysis: (**a**) survey scan, high resolution deconvoluted (**b**) C1 & (**c**) O1s spectra for nettle leaves 1:1 CNS.

Elemental analysis was also carried via X-ray photoelectron spectroscopy (XPS) on a representative nettle CNS sample (nettle leaves 1:1), which confirmed the existence of C as major element with O and N as minor elements and Si as a trace element in the prepared CNS (Fig. 3).

In conclusion, the EDS and XPS analyses reveal that C is the major element in the nettle-derived CNS. The presence of O is also confirmed, which is common in biomass-derived C^34^. These analyses further confirm the absence of any metal impurities in the nettle-derived CNS.

### TGA of CNS

**Figure 4:**
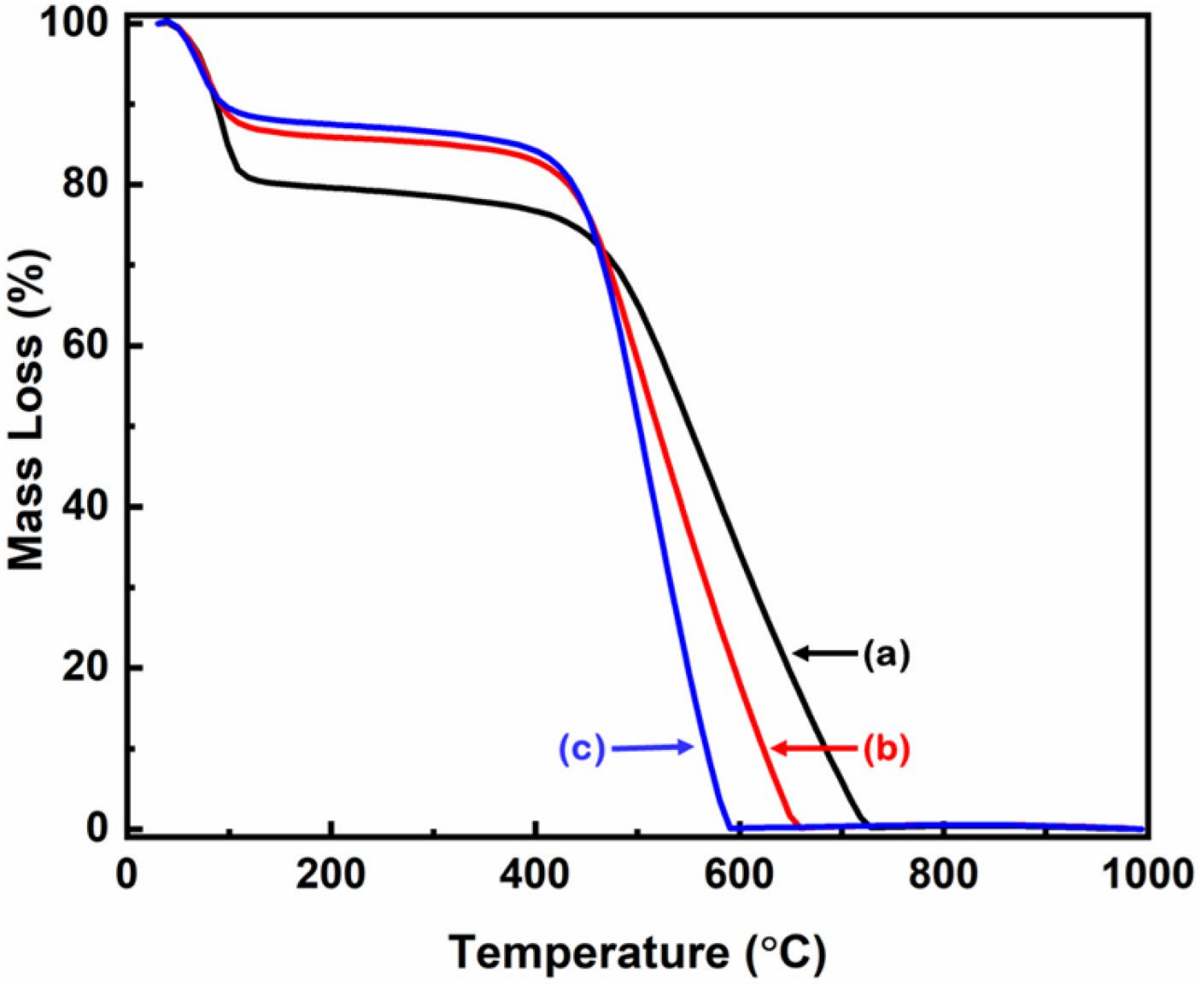
The TGA curves of CNS prepared at 650 °C from nettle (**a**) stems and (**b**,**c**) leaves using NaHCO_3_ as activating agent. The ratio of nettle powder and NaHCO_3_ is 1:1 (**a**,**b**) and 1:2 (**c**).

The thermogravimetric analysis (TGA) analysis was performed in air to investigate the thermal decomposition of the prepared CNS. The TGA curves shown in Fig. 4 demonstrate that the mass loss occurred in two steps. In the first step, the mass loss occurred in the temperature range of 40 °C to 100 °C, which is mainly due to the evaporation of the moisture and residual water molecules. In the second step, the major mass loss was observed between 400 °C and 700 °C in all the three samples, showing the start of thermal decomposition at around 400 °C and stabilizing at around 700 °C. Another aspect worth being highlighted is the final mass: above 700 °C, all the prepared CNS resulted in complete decomposition (i.e. zero mass) which indicates the preparation of metal-free CNS by this process^35^.

### Diffraction pattern of CNS

**Figure 5:**
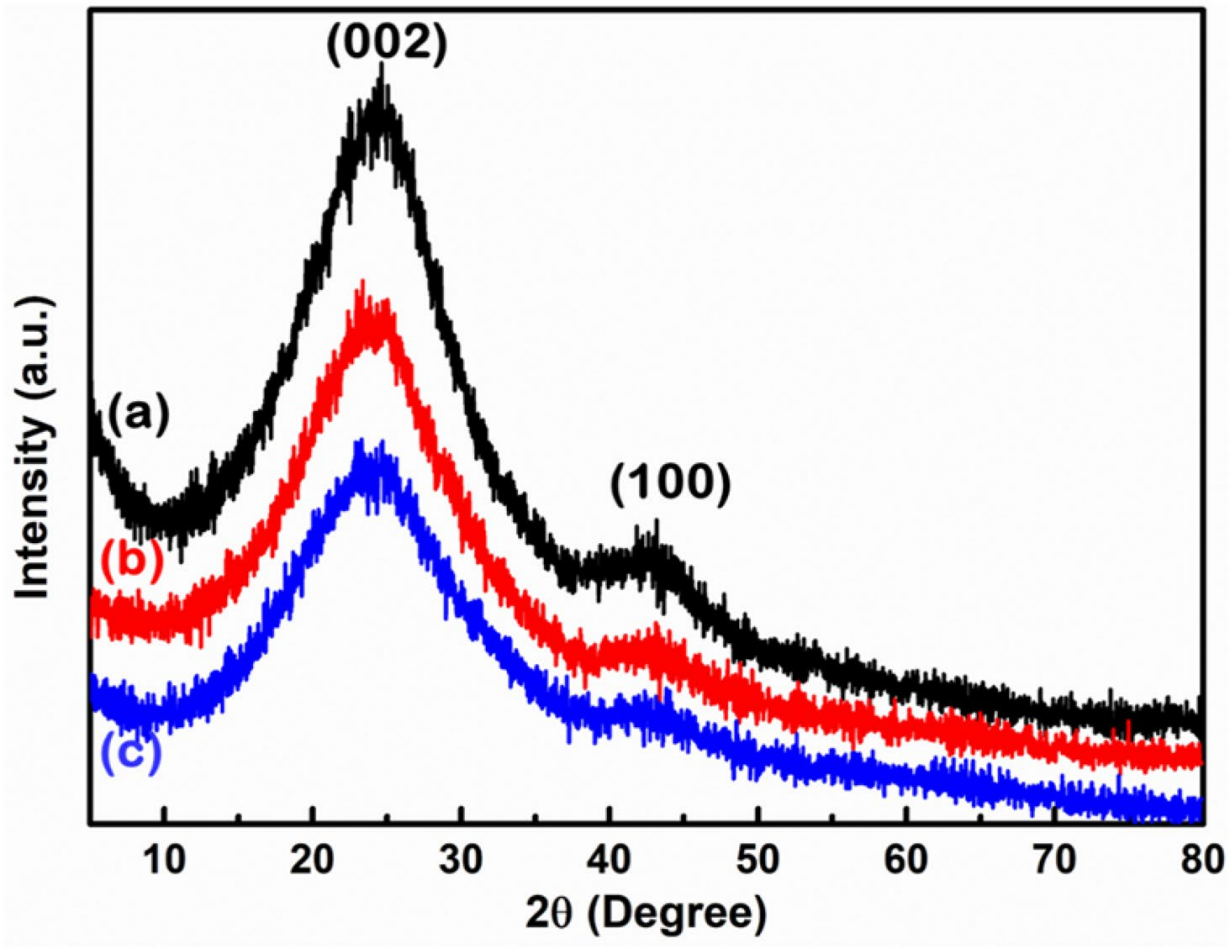
XRD spectra of CNS prepared at 650 °C from nettle (**a**) stems and (**b**,**c**) leaves using NaHCO_3_ as activating agent. The ratio of nettle powder and NaHCO_3_ is 1:1 (**a** and **b**) and 1:2 (**c**).

XRD was used to investigate the diffraction nature and phase formation of the prepared CNS. Figure 5 shows the XRD patterns of nettle-derived CNS prepared with different activation processes, i.e. (a) CNS1:1 (nettle stems: NaHCO_3_), (b) CNS1:1 (nettle leaves: NaHCO_3_) and (c) CNS1:2 (nettle leaves: NaHCO_3_). All the samples show identical diffraction patterns and all the peaks are well consistent with the pure graphitic C. The XRD spectra of the activated CNS show two peaks: the first characteristic broad peak at around 2θ = 24° is related to C(002) diffraction peak, which corresponds to the reflection of graphitic C and a second less intense, broader peak at around 2θ = 42° is assigned to C(100) diffraction peak, which accounts for amorphous C (JCPDF: 41–1487)^36,37^. No additional peak from the minor trace elements could be observed in the XRD spectra, due to their relatively low concentration, suggesting the formation of pure CNS.

### Raman Spectroscopy

**Figure 6:**
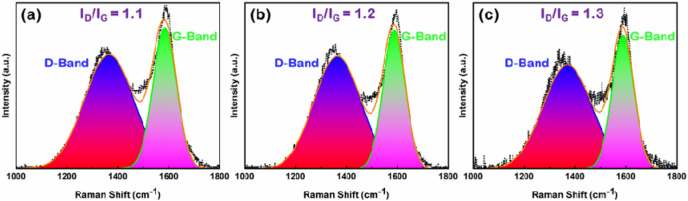
Raman spectra of CNS prepared at 650 °C from nettle leaves (**a**,**c**) and (**b**) stems using NaHCO_3_ as activating agent. The ratio of nettle powder and NaHCO_3_ is 1:1 (**a**,**b**) and 1:2 (**c**).

Raman spectroscopy was used to investigate various disordered domains and the graphitization degree of the prepared nettle-derived CNS. The Raman spectra show two peaks, i.e. the G-band and D-band (Fig. 6), with the latter accounting for disorder and the former for perfect graphite crystals^38^. Typically, the G band centered at 1570 cm^−1^ corresponds to the two dimensional in-plane motion of strongly bonded sp^2^ C atoms, highlighting a characteristic of graphitic C and the D band centered at 1340 cm^−1^ relates to the defect sites or disordered tetrahedral sp^3^-hybridized C atoms^39,40^. The ratio of integrated intensities of D band and G band (I_D_/I_G_) represents the number of defects in the structure^38^. The ratios of areas under D-band and G-band was used to calculate I_D_/I_G_^41^. The calculated I_D_/I_G_ ratios are 1.2, 1.1 and 1.3 for nettle stem-derived CNS1:1, nettle leaf-derived CNS1:1 and CNS 1:2, respectively. This high intensity ratios entail the existence of disordered C in the prepared porous CNS^2^. These results suggest that the I_D_/I_G_ ratio of nettle leaf-derived CNS1:1 is lowest, i.e. this samples possess the lowest number of defects^42^. This means that the highest degree of graphitization is found in NaHCO_3_-activated nettle leaf-derived CNS1:1.

The high graphitization degree of the nettle stem-derived CNS is due to the structure of the tissues: just like hemp^3^, the stem of nettle also contains bast fibres (Supplementary Fig. 1), that are long elements with a thick cellulosic wall (called gelatinous, or G-layer). Bast fibres are indeed characterized by crystalline cellulose and are hypolignified^43^. There are however differences between hemp and nettle bast fibres. The latter do not show labelling when the LM10 antibody recognizing the hemicellulose xylan is used and display a different structure of the G-layer^18^ compared to hemp^44^. At the transmission electron microscope (TEM), the G-layer of nettle bast fibres appears indeed less compact than that of hemp, with the formation of local loose regions where the cellulosic layer seems to be flaking off (Supplementary Fig. 1c)^18^. Such a loose structure may facilitate the process of CNS preparation by favouring disassembly of the layers and penetration of the activating agent.

### Nitrogen adsorption–desorption isotherms and corresponding BJH pore-size distributions

**Figure 7:**
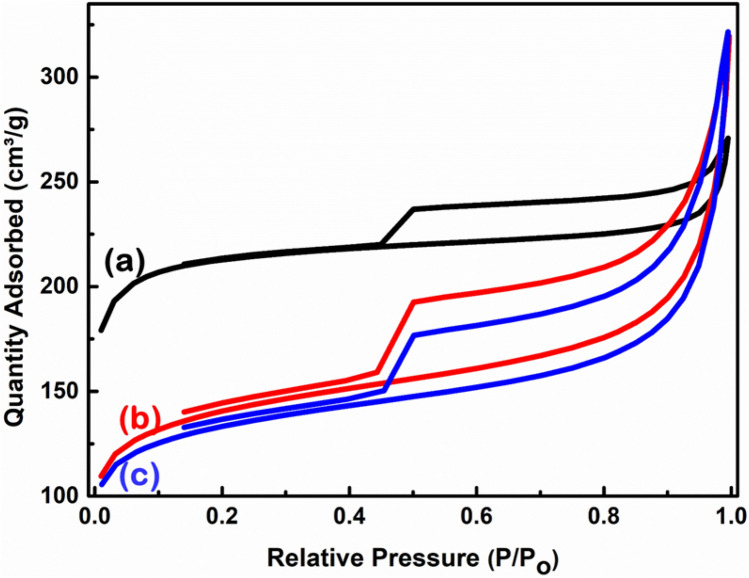
BET Isotherms of CNS prepared at 650 °C from nettle (**a**) stems and (**b**,**c**) leaves using NaHCO_3_ as an activating agent. The ratio of nettle powder and NaHCO_3_ is 1:1 (**a**,**b**) and 1:2 (**c**).

The pore-network structure and Brunauer–Emmett–Teller (BET) specific surface area for the prepared nettle-derived CNS were investigated based on the physical nitrogen adsorption–desorption analysis. High surface area and combined pores containing C samples are very important for improved biological applications. NaHCO_3_ was used as an activating agent to improve the porous structure and enhance the BET specific surface area of the prepared CNS. The nitrogen adsorption isotherms of the prepared samples are shown in Fig. 7 and described according to the IUPAC report^45^. It can be clearly seen that all the three samples show similar type-IV isothermal sorption curves, due to the presence of hysteresis loops^45–47^. The initial isotherm curvatures of the prepared C samples are assigned to micro- and mesopores, while the ascending curvature of the plateau (type H3 loop) in the high relative pressure range (P/P_0_ = 0.85 − 1.00) is inherent to macropores^45–47^. The wider and higher ascending curvature in the CNS prepared from leaves indicates the presence of more macropores than the CNS prepared from the stems. This more pronounced adsorption property in the isotherms of CNS from leaves is assigned to an enhanced N_2_ uptake associated with micropore filling.

**Figure 8:**
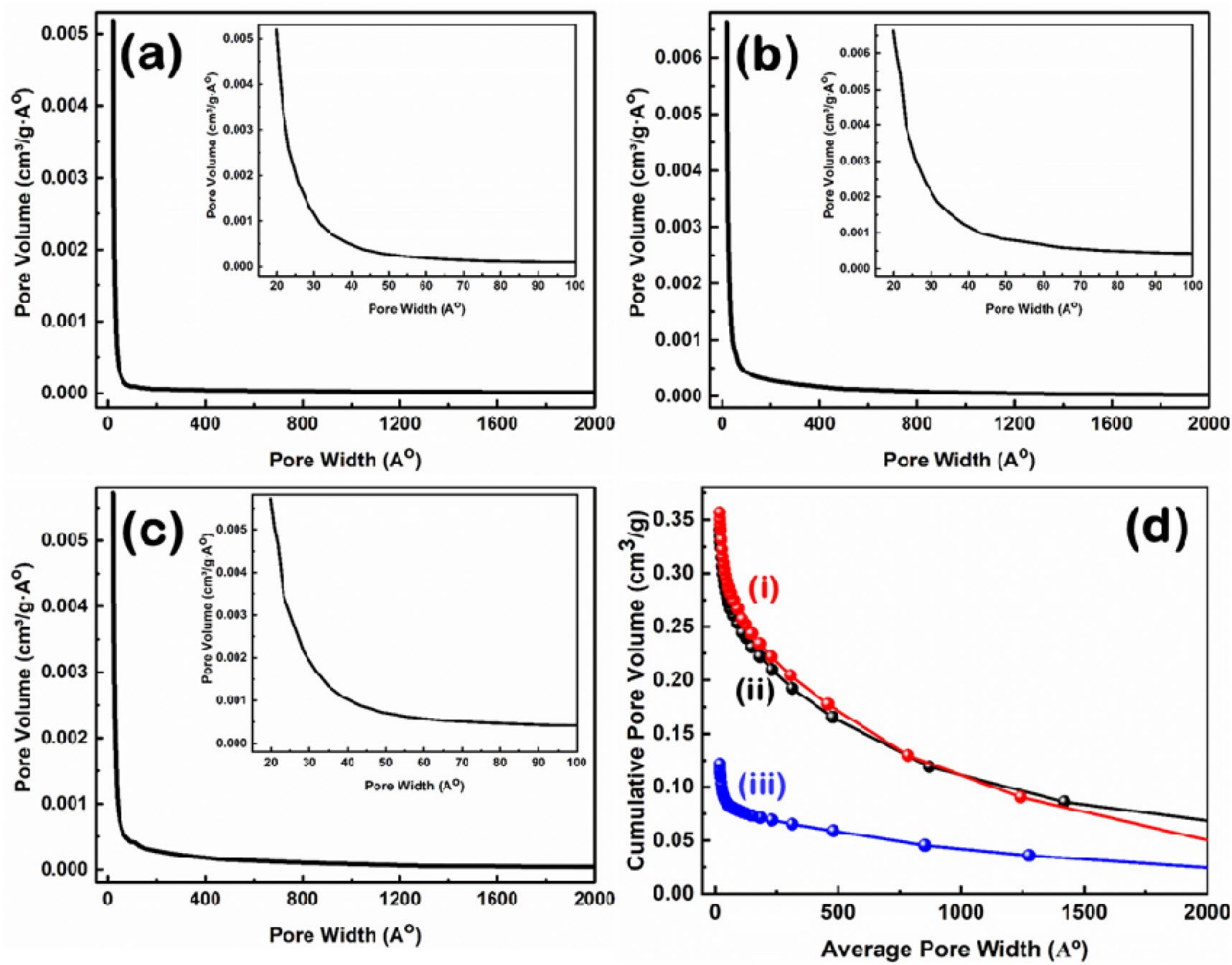
BJH pore size distribution of CNS prepared at 650 °C from nettle (**a**) stems and (**b**,**c**) leaves using NaHCO_3_ as an activating agent. The ratio of nettle powder and NaHCO_3_ is 1:1 (**a** and **b**) and 1:2 (**c**). Insets show the corresponding low pore width zone. (**d**) The corresponding cumulative pore size distribution of nettle leaf- (i and ii) and nettle stem- (iii) derived CNS with the ratio for nettle powder and NaHCO_3_ of 1:1 (ii and iii) and 1:2 (i).

The corresponding Barrett-Joyner-Halenda (BJH) pore size distribution of the prepared samples is shown in Fig. 8(a–c), while the structure properties of the prepared CNS using different ratios of the nettle powder and NaHCO_3_ are summarized in Table 1. The corresponding cumulative pore size distribution based on the BJH method is shown in Fig. 8d.

**Table 1:** BET surface area, average pore volume and average pore diameter of the prepared nettle-derived CNS at 650 °C, using different NaHCO_3_: tissue powder ratios.

Samples	Biomass sources	BET surface area (m^2^/g)	Average pore volume (cm^3^/g)	Average pore diameter (Å)
CNS1:1	Nettle stems	718	0.12	20.1
CNS1:1	Nettle leaves	482	0.34	31.7
CNS1:2	Nettle leaves	478	0.36	32.2

The two samples of CNS prepared using nettle leaves have a relatively similar BET specific surface area, while the CNS prepared using nettle stems show significantly higher value than the leaf samples (Table 1). The average pore volume and pore diameter of the CNS prepared from nettle leaves are larger than the CNS prepared from stems. This observation can be explained by the presence of a high number of macropores in the CNS prepared from nettle leaves, while the additional micropores formation in the CNS prepared from stems contribute to the improvement of the BET specific surface area^48^. As shown in Table 1, CNS1:1 prepared from nettle stems show the highest BET surface area of 718 m^2^⁄g, while CNS1:1 and CNS1:2 have BET surface areas of 482 m^2^/g and 478 m^2^/g, respectively. The BET specific surface area is in the same range as the one previously calculated for cornstalks-derived C nanomaterial (between 320–790 m^2^⁄g)^4^, while the thickness is in agreement with the values reported for CNS prepared from hemp (10–30 nm)^3^, tal palm (22 nm)^2^, waste coffee ground (ca. 20 nm)^1^. Hence, nettle leaves and stems are suitable renewable resources for the preparation of CNS with the advantage of providing very homogeneous material, since the plants used are clones, as well as a higher amount of fibres compared to wild nettle.

### Effects of nettle-derived CNS on the germination of tobacco pollen

Evaluating the environmental impact of either the production or utilization of a new material (such as CNS) implies a complex system of analysis requiring the contribution of different, but complementary, scientific disciplines. In plants, C nanomaterials negatively impact germination in both monocots and dicots. For example, in rice, graphene at 50 µg/mL delayed by three days the germination of the seeds compared to the control group and also caused a decrease in moisture content, probably due to the physical blocking of the seed coat pores and consequent water uptake impairment^49^. Recently, a study focused on an economically-important crop, i.e. tomato, showed that while C nanotubes (single-walled and multi-walled) in soil delayed early growth and flowering, they did not affect later developmental stages^50^. Single-walled nanotubes, however, caused an increase in salicylic acid, a finding denoting the presence of a stress response in the plants which is not present in the case of multi-walled tubes^50^.

The germination of tomato seeds primed with C nanomaterials was not affected; however, parameters such as root length and hypocotyl biomass were impaired, while chlorophyll content, as well as vitamin C, β-carotene, phenols and flavonoids increased^51^. These results show that C nanomaterials can be used as biostimulants, but their stimulating effects should be carefully determined beforehand to establish the right concentration for the crop examined.

As a first step towards the study of the effects of C nanomaterials on plants, it is possible to use a simplified system consisting in cell models that respond quickly to environmental stressors^52,53^. Obviously, such analyses do not allow an overall assessment of the environmental impact of a material or technology, but they can provide an initial indication of their toxic potential. Many model cells can be used in such an assessment, including the pollen tube, because it is a critical structure during plant reproduction^54,55^. One of the most important reasons for selecting the pollen tube as a model cell is that the evaluation of toxic effects can be quantified in a relatively simple way. In fact, the pollen tube is a cell that grows linearly and in which many cytological events follow one another in a precise spatial–temporal order. These characteristics make the pollen tube a model cell system suitable for providing measurable data^56^.

Pollen is very sensitive to environmental pollutants compared to other plant cells and this allows to assess the impact of a wide range of chemicals on plant metabolism^57,58^. Therefore, it is used as an indicator of air pollution because the performance of pollen (described in terms of germination and elongation of pollen tubes) is affected by airborne gas pollutants. Atmospheric particulate matter also affects pollen performance, for example Ag and Pd nanoparticles impair in vitro pollen germination and pollen elongation in kiwi^59,60^. Graphene oxide nanoparticles can also have a negative effect on the germination and growth of tobacco and hazel pollen tubes^19^.

In Supplementary Fig. 2, images from inverted microscopy and SEM are shown. CNS tend to form aggregates, especially at the highest concentration (Supplementary Fig. 2a–c), rather than evenly distribute in the medium. This is confirmed by SEM observations at low resolution, where the aggregates formed are well visible (Supplementary Fig. 2d–f).

In the presence of pollen grains, the CNS clusters tend to adhere to the pollen grains (Supplementary Fig. 3).

It was previously suggested that the pollenkitt of *N. tabacum* pollen grains could act as a matrix favouring the adsorption of CNS^19^. The presence of CNS aggregates sometimes made it difficult to correctly visualize the pollen and therefore to evaluate the germination percentage.

**Figure 9:**
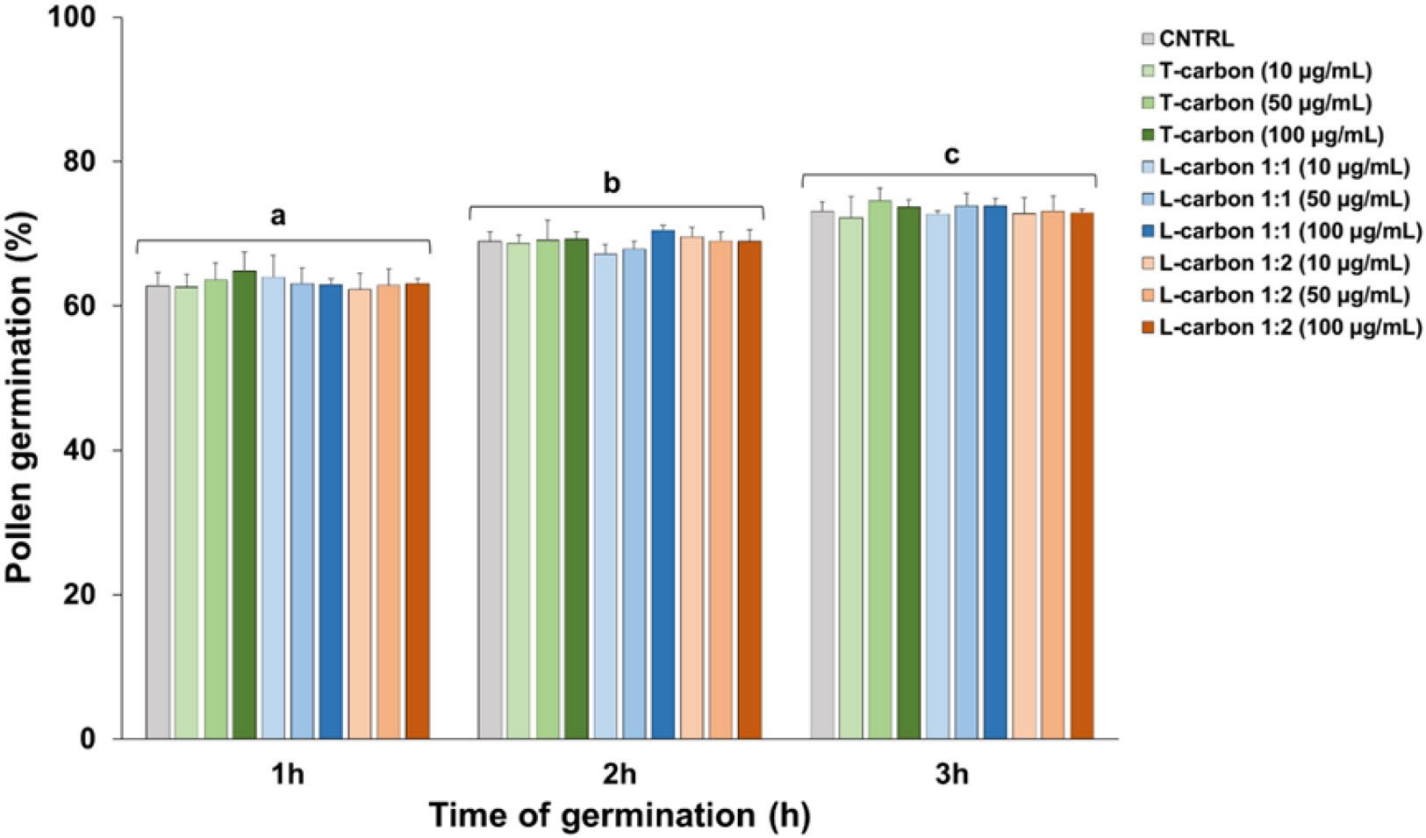
Effects of increasing concentrations (10–50-100 µg/mL of BK_suc_) of CNS on the germination of pollen grains from *N. tabacum*. The measurements are relative to the control (CNTRL) and comprise a total treatment time of 3 h. The values are reported as means ± standard deviation (n = 150). A two-way ANOVA was conducted to examine the effect of time and different CNS concentration on pollen germination. There was a statistically significant interaction of time on germination, F (2, 60) = 252.460, *p* = 0.000. Different letters denote statistically significant changes among groups at the two-way ANOVA followed by Tukey’s post-hoc test.

As shown in Fig. 9, the percentage of germinated pollen increases slightly from 1 to 3 h of growth. From personal experience, a value above 50% is normally considered as acceptable in the case of frozen pollen. Germination data are thus consistent with what is usually observed. Data in Fig. 9 also show that the presence of stem- and leaf-derived CNS has no effect on pollen germination at any of the three concentrations tested for a total germination time of 3 h. Therefore, even if CNS form aggregates around pollen grains, this has no impact on pollen germination. As can be seen in Fig. 9, CNS do not affect pollen growth, even at the maximum concentration. Therefore, nettle-derived CNS have no inhibitory effects on tobacco pollen germination. After all, the germination of pollen grains is only one of the processes that take place during the reproduction of seed plants. Therefore, this result is not surprising because the possible negative effects of CNS could affect other steps, such as pollen activation, or pollen tube growth^19^, or specific molecular events^22^. Future studies, focused on the further development of pollen tubes, will clarify the possible effects of CNS during other processes related to pollen tubes such as, for example, the transport of sperm cells or a possible inhibitory effect of nanocomposites *in planta* i.e. on the adhesion, activation and growth of the pollen tube on stigma and style.

### Effects of the nettle-derived CNS on the growth of *P. subcapitata*

The evaluation of the ecotoxicological effects of nettle CNS was complemented by tests on the green microalgae *P. subcapitata*. This is a phytoplanktonic freshwater aquatic model for ecotoxicological investigations and is widely used for studies addressing the interaction of nanoparticles with microalgae cells in aquatic environments^61,62^.

In the literature, several studies have investigated the effects of C nanomaterials on microalgae and shown a higher toxicity in the case of oxidized nanomaterials. Oxidation occurs as a spontaneous aging/weathering process after the nanomaterials are released in the environment. *Chlorella pyrenoidosa* increased the antioxidant response as a result of exposure to oxidized multi-walled C nanotubes, with enhanced pentose phosphate pathway, cell division and formation of polyphosphate bodies^63^. However, when these protective responses were overwhelmed, the algae suffered from toxicity caused by membrane damage and denaturation of macromolecules. Oxidized C nanotubes adsorbed to the cell surface and penetrated via puncture and subsequent endocytosis^63^.

**Figure 10:**
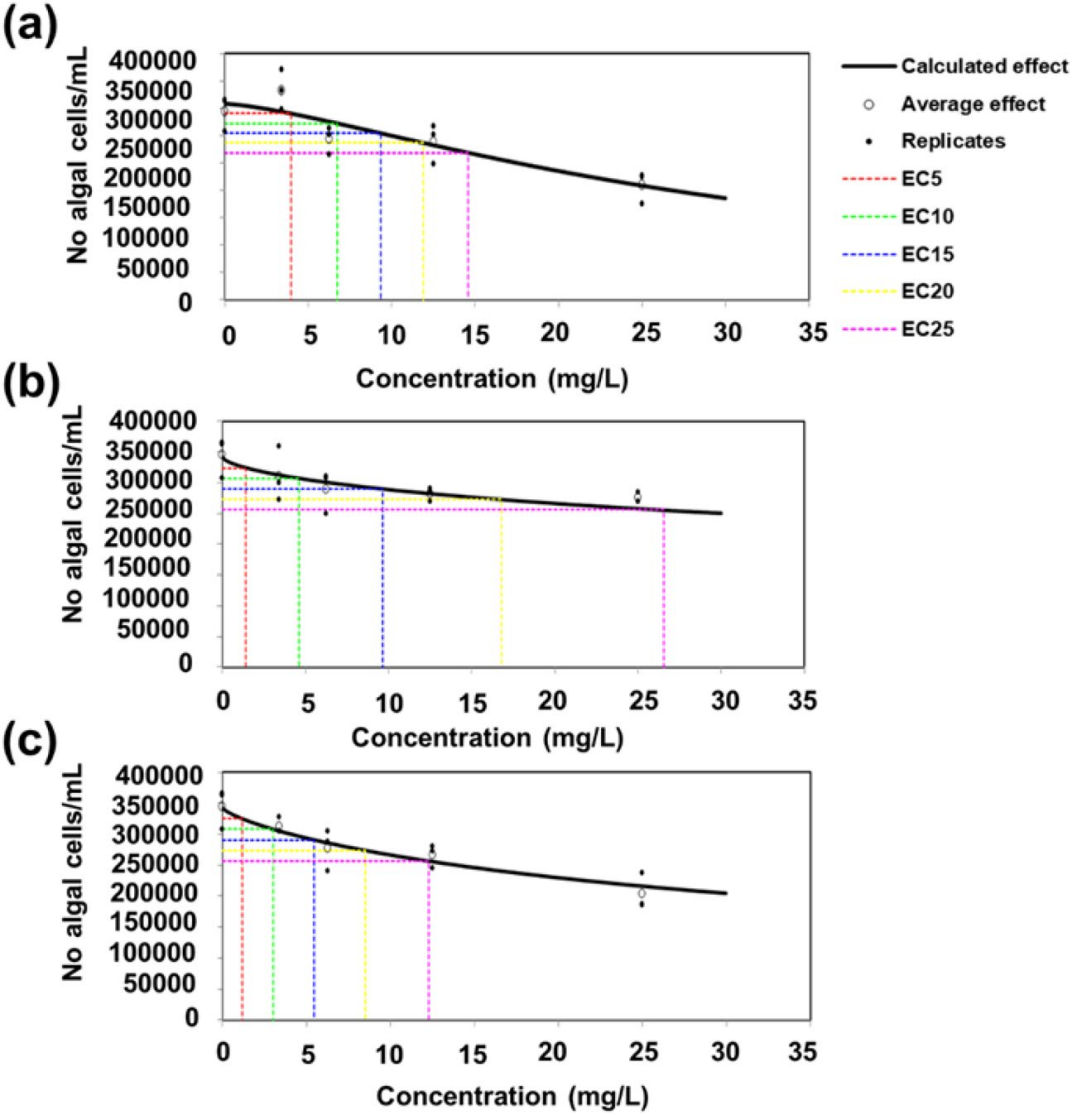
Inhibitory effect of nettle CNS at different concentrations on algal cell number after 72 h. CNS from stem 1: 1 (**a**), CNS from leaves 1:1 (**b**), CNS from leaves 1:2 (**c**).

Different concentrations (3.4–6.25–12.5 and 25 µg/mL) were tested and the number of algal cells was counted after 72 h of growth. As it can be observed in Fig. 10, at higher concentrations, the nettle CNS cause a decrease in the number of *P. subcapitata* cells for the stem- and leaf-derived CNS prepared using a ratio NaHCO_3_: nettle powder equal to 1:2. The nettle CNS tend to form aggregates (Supplementary Fig. 2) and the accumulation of C nanomaterials around algal cells was reported to cause shading and, subsequently, to reduce light availability thus resulting in growth impairment^64,65^.

The toxicity of C nanomaterials is due to different physical parameters, i.e. size, surface charge and area^66^. A higher surface area could adsorb more transition metals and ultimately trigger the formation of more reactive oxygen species (ROS) via Haber–Weiss and Fenton-like reactions^67^. The BET surface area for the leaf CNS is, however, the same, irrespective of the ratio of activating agent used (Fig. 7 and Table 1).

The toxicity of C nanomaterials in algae can also be due to the presence of heavy metal impurities due to low-efficiency manufacturing process (which, for the lowest manufacturing processes, can constitute up to 99.9% of the total production output^68^). A recent study has shown that metal impurities in C nanotubes affect the esterase activity of the algae *Attheya ussuriensis*, *Chaetoceros muelleri*, *Heterosigma akashiwo* and *Porphyridium purpureum* (with the latter showing the lowest changes)^69^. It should however be noted that, in the present study, no metal impurities were detected (Figs. 2 and 3).

The higher inhibitory impact on growth by the nettle leaf-derived CNS1:2 may also be ascribed to its high defective nature (higher I_D_/I_G_, lower graphitization) and smaller average sheet thickness with a higher adsorption average pore width. Such properties of leaf-derived CNS1:2 facilitate the easy absorption at higher concentrations and thus cause a decrease in the number of *P. subcapitata* cells. Although the EC_25_ value of leaf CNS1:2 is higher, it should however be noted that all the EC_25_ values reported for the nettle CNS lie within the same order of magnitude; thereby, no statistically significant differences are visible between the CNS. The values in Table 2 report the various essential parameters that may contribute to the toxicity of nettle C nanomaterials on microalgal cells.

**Table 2:** Comparison of surface and structural parameters of the nettle-derived CNS for the effect toxicity using *P. subcapitata*. EC_25_ (mg/L, with the 95% confidence intervals between parentheses) are indicated for comparative purposes.

Parameter	Stem CNS	Leaf CNS1:1	Leaf CNS1:2
I_D_/I_G_	1.2	1.1	1.3
CNS average thickness	36 nm	32 nm	27 nm
Adsorption average pore width	20.1 Å	31.7 Å	32.2 Å
BJH adsorption average pore width	70.8 Å	98.7 Å	111.2 Å
EC_25_	14.6 (8.2 21.8)	26.5 (16 46)	12.3 (7.2 20.3)

## Conclusions

The preparation of highly porous activated CNS from nettle tissues with interesting properties in terms of porosity and active surface area was demonstrated in the present study by using pyrolysis at 650 °C. The stems and leaves of *U. dioica* clone 13 were used as raw material and NaHCO_3_ as activating agent. Various ratios of the nettle powder and NaHCO_3_ were mixed and carbonized together under N_2_ environment to get the porous CNS. The C obtained from this process showed high porosity and BET specific surface area. The highest BET specific surface area (718 m^2^/g) was obtained from the nettle stem-derived CNS, which is in the range of commercially available activated C.

The potential ecotoxicological effects of nettle CNS were evaluated on tobacco pollen and the green microalga *P. subcapitata*. Tobacco pollen germinated both under control conditions and in the presence of progressively increasing concentrations of CNS; under the experimental conditions used, CNS formed dark aggregates that were easily observable as their concentration increased. The presence of such dense aggregates forming a sheath around the algal cells and reducing light availability is probably the reason for the observed growth inhibition. The growth inhibition for algae was however associated to high concentrations of the CNS. The strongest inhibitory effects on the growth of *P. subcapitata* were observed for leaf CNS1:2, a finding suggesting that the low degree of graphitization has a negative effect on algal growth.

By using a type of biomass that can be propagated rapidly by stem cuttings and in large amounts, highly porous CNS can be produced at a large scale and low cost for useful industrial applications. Overall, the work here presented is novel because it shows an additional way to valorize a neglected species considered a weed. The advantage of nettle over other plants is its fast growth and propagation using stem cuttings, which ensures homogeneous starting raw material.

## Materials and methods

### Growth of plants and chemicals

The whole stems and leaves of stinging nettle (*U. dioica* clone 13) were collected from 1.5 months-old plants grown under controlled conditions^17^. The clone used produces a higher yield of bast fibres (16%) compared to wild nettle (ca. 4–5%) and it was bred by G. Bredemann between 1927–1950 at the Institute of Applied Botany in Hamburg^70^. Sodium bicarbonate (NaHCO_3_) and hydrochloric acid (HCl) were obtained from Sigma-Aldrich. The nitrogen (N_2_) gas with a purity of 99.99% was purchased from SCG gas supply center, Jubail, Saudi Arabia. De-ionized (DI) water was obtained from a water purification system (Barnstead Nanopure, Thermo Scientific, USA).

### Preparation of CNS

CNS were prepared by pyrolysis of the nettle stems and leaves using NaHCO_3_ as an activating agent. The stems and leaves were collected, washed with DI water and then dried in an electric oven at 100 °C for 24 h. The dried stems and leaves were separately crushed by a high-precision grinding machine to obtain a very fine powder. Powder (particle size ≤ 100 µm) was collected after passing it through a 100 µm mesh. Three different samples were prepared by mixing the obtained powder with different ratios of NaHCO_3_. The ratio of nettle stem powder and NaHCO_3_ was 1:1 (referred to as CNS1:1), while for the leaves and NaHCO_3_ the mass ratios were 1:1 and 1:2 (referred to as CNS1:1 and CNS1:2, respectively). The final uniform mixture was then placed in an alumina crucible and carbonized at 650 °C for 5 h at a heating and cooling rate of 10 °C/min and 5 °C/min, respectively, in a tube furnace under the protection of nitrogen flow. For the last step, the samples were placed in glass beakers containing 0.5 M HCl and ultrasonicated for 2 h and then washed two times with DI water. Following this step, the obtained samples were then filtered through a filter paper. Finally, the filtered samples were dried in an electric oven at 60 °C for 24 h to obtain the nettle-derived CNS.

### Characterization of CNS

The morphology of the prepared samples was analyzed using FESEM (TESCAN LYRA 3, Czech Republic). The FESEM was operated at 20 kV. EDS data were recorded with an Oxford Instruments Xmass detector, equipped with the FESEM and analyzed with the LINK INCA program system.

The TGA of CNS were recorded by the thermal analyzer (TGA1 STAR^e^ SYSTEM), having a temperature range from room temperature up to 1000 °C. An X-ray monochromator (ESCALAB 250Xi XPS Microprobe, Thermo Scientific, USA) was utilized to record the XPS of the prepared CNS. An X-ray diffractometer, Rigaku Ultima (high-resolution) equipped with Cu-K (alpha) radiation was used to obtain the XRD patterns of the prepared CNS. The graphite structure of the synthesized CNS was detected and studied by using a Raman spectrometer, iHR320 with CCD detector (HORIBA), equipped with green laser (300 mW) having an excitation wavelength λ_o_ = 532 nm. For the measurement of the surface area and pore size of the prepared CNS, BET and BJH analyses were performed by using Micromeritics ChemiSorb 2750. A high-precision grinding machine was used to make a fine powder and a Power Sonic 603 ultrasonic cleaner was used for sonication.

### Optical and transmission electron microscopy (TEM) of nettle stem tissues

The preparation of nettle stem tissue sections and Toluidine Blue O staining were performed as previously described^17,71^. Nettle bottom internodes (5 mm thickness, localized at the stem base) were fixed in glutaraldehyde/paraformaldehyde/caffeine (1%/2%/1% v/v in Milli-Q water) under vacuum for 15 min and o/n at 4 °C, dehydrated in an ethanol series (70–95–100%), impregnated in resin containing PEG 400 (2% v/v) and dimethacrylate ethylene glycol (0.4% w/v) and finally included in Technovit 7100 resin (Technovit 7100, Heraeus Kulzer GmbH, Hanau, Germany). Cross sections of 10 μm thickness were prepared using a microtome and stained with 0.05% w/v Toluidine Blue O.

Sample processing for immunoTEM and detection of crystalline cellulose with CBM3a were as previously reported^18^. Briefly, the bottom internode was sampled by using a clean razor blade and thereafter fixed for 2 h at room temperature (RT) and o/n at 4 °C in 2% v/v glutaraldehyde and 1.6% v/v paraformaldehyde in 0.1 M phosphate buffer pH 6.9. Samples were rinsed with phosphate buffer two times for 10 min each, then dehydrated in a graded series of ethanol. Samples were infiltrated with LR‐White resin at 1:1 ratio with ethanol to pure resin. After polymerization for 48 h at 40 °C, ultrafine sections were obtained with a diamond knife and the ultramicrotome LKB NOVA. The sections were collected on gold grids and blocked for 20 min with normal goat serum diluted 1:30 in dilution buffer (0.05 M Tris‐HCl pH 7.6, 0.9% w/v NaCl and 0.2% w/v BSA). Sections were incubated with the CBM3a protein^72^ at 5 μg/mL at RT for 1.5 h in conjunction with the mouse monoclonal anti‐His antibody diluted 1:100 in dilution buffer + 0.1% v/v Tween 20 for 1.5 h. Samples were washed and incubated with the anti‐mouse antibody conjugated with 10 nm gold particles for 45 min at RT. Sections were visualized with the Philips MORGAGNI 268 80 kV transmission electron microscope, equipped with MEGAview II camera and elaborated with the Analysis software.

### Pollen grains’ germination assay and microscopy

Pollen grains of tobacco were obtained from plants grown at the Botanical Garden of the University of Siena (Italy). For analysis, the pollen was used after having been stored at -20 °C. The pollen was germinated in BK germination medium with 12% sucrose (referred to as BK_suc_)^73^. Germination time was usually 2–3 h. In the case of CNS treatment, the nanosheets were added to the growth medium at different concentrations (10, 50 or 100 µg/mL) and the pollen tube growth was monitored exactly as for the control. CNS were initially sonicated to facilitate dispersion, after that they were added to the growth medium. Germination tests were carried out in 24-well plates with pollen at the concentration of 1 mg/mL. At different time points, pollen tubes were observed with an optical microscope (Zeiss AxioPhot, 10X objective, MRm videocamera). The germination of pollen grains was evaluated by observing 150 pollen grains for each condition. For a statistical evaluation of the effects of CNS at different concentrations on pollen germination, a two-way ANOVA was applied after checking normality with the Shapiro–Wilk test and homogeneity with the Levene’s test (SPSS Statistics v19 IBM SPSS, Chicago, IL, USA).

### *P*. *subcapitata* growth in the presence of nettle CNS

The growth inhibition test was performed with the green algal species *P. subcapitata* (CCAP 278/4, Scotland, United Kingdom). The test is a modification of the OECD 201 standard^74^ in a 12-well plate format. A pre-culture of algae was started 3 days before the beginning of the experiment and algae in exponential growth phase were used. The OECD medium^74^ (FUJIFILM Wako Pure Chemical Corporation) was used in order to prepare the test concentrations, which were inoculated with exponentially growing algae in a concentration of 10^5^ cells/mL. One mL of algae with or without the different nettle CNS at increasing concentrations (3.4–25 mg/L) were placed in the wells of 12-well plates and were continuously shaken at 50 rpm (Gerhardt Analytical Systems, Germany) under a cool white fluorescent light (46.2 mmol m^-2^ s^-1^) and temperature 20 °C ± 1 °C. Algal suspension with the highest concentration of the carrier solvent (2.5% v/v DMSO) was used as negative control. Three replicates per concentration and for the control were used. After 72 h, the cell concentration was determined manually in a Bürker-Türk haemocytometer. Growth rates were calculated as indicated in the guideline and the results were expressed as % inhibition of growth rate relative to the untreated control.

## Supplementary Information

Supplementary Information

## Data Availability

All data generated or analyzed during this study are included in this published article and the supplementary material.

## References

[1] Yun YS (2015). Hierarchically Porous Carbon Nanosheets from Waste Coffee Grounds for Supercapacitors. ACS Appl. Mater. Interfaces.

[2] Ahammad AJS (2019). Porous tal palm carbon nanosheets: preparation, characterization and application for the simultaneous determination of dopamine and uric acid. Nanoscale Adv..

[3] Wang H (2013). Interconnected Carbon Nanosheets Derived from Hemp for Ultrafast Supercapacitors with High Energy. ACS Nano.

[4] Wang L (2013). Porous graphitic carbon nanosheets derived from cornstalk biomass for advanced supercapacitors. Chemsuschem.

[5] Li J, Liu W, Xiao D, Wang X (2017). Oxygen-rich hierarchical porous carbon made from pomelo peel fiber as electrode material for supercapacitor. Appl. Surf. Sci..

[6] Chen X (2017). A novel hierarchical porous nitrogen-doped carbon derived from bamboo shoot for high performance supercapacitor. Sci. Rep..

[7] Hao E (2017). Rich sulfur doped porous carbon materials derived from ginkgo leaves for multiple electrochemical energy storage devices. J. Mater. Chem. A.

[8] Chang J (2015). Activated porous carbon prepared from paulownia flower for high performance supercapacitor electrodes. Electrochim. Acta.

[9] Yang H, Ye S, Zhou J, Liang T (2019). Biomass-Derived Porous Carbon Materials for Supercapacitor. Front. Chem..

[10] Kumar S, Yadav A, Yadav M, Yadav JP (2017). Effect of climate change on phytochemical diversity, total phenolic content and in vitro antioxidant activity of Aloe vera (L.) Burm.f.. BMC Res. Notes.

[11] Xu J (2019). Response of Bioactive Phytochemicals in Vegetables and Fruits to Environmental Factors. Eur. J. Nut. Food Saf..

[12] Liu M, Liu G, Gong L, Wang D, Sun J (2014). Relationships of Biomass with Environmental Factors in the Grassland Area of Hulunbuir China.. PLoS ONE.

[13] Joshi R, Singla-Pareek SL, Pareek A (2018). Engineering abiotic stress response in plants for biomass production. J. Biol. Chem..

[14] Farag MA, Weigend M, Luebert F, Brokamp G, Wessjohann LA (2013). Phytochemical, phylogenetic, and anti-inflammatory evaluation of 43 *Urtica* accessions (stinging nettle) based on UPLC-Q-TOF-MS metabolomic profiles. Phytochemistry.

[15] Xu X (2019). Insights into Lignan Composition and Biosynthesis in Stinging Nettle (*Urtica dioica* L.). Molecules.

[16] Guerriero G (2018). Production of Plant Secondary Metabolites: Examples, Tips and Suggestions for Biotechnologists.. Genes (Basel).

[17] Backes A (2018). Sucrose synthase gene expression analysis in the fibre nettle (*Urtica dioica* L.) cultivar “clone 13”. Ind. Crops Prod..

[18] Xu X (2019). Cell wall composition and transcriptomics in stem tissues of stinging nettle (*Urtica dioica* L): Spotlight on a neglected fibre crop. Plant Direct.

[19] Carniel FC (2018). Graphene oxide impairs the pollen performance of *Nicotiana tabacum* and *Corylus avellana* suggesting potential negative effects on the sexual reproduction of seed plants. Environ. Sci. Nano.

[20] Begum P, Ikhtiari R, Fugetsu B (2011). Graphene phytotoxicity in the seedling stage of cabbage, tomato, red spinach, and lettuce. Carbon.

[21] Anjum NA (2014). Single-bilayer graphene oxide sheet impacts and underlying potential mechanism assessment in germinating faba bean (*Vicia faba* L). Sci. Total Environ..

[22] Candotto Carniel F (2020). Beyond graphene oxide acidity: Novel insights into graphene related materials effects on the sexual reproduction of seed plants. J. Hazard. Mater..

[23] Wu K (2016). Large and porous carbon sheets derived from water hyacinth for high-performance supercapacitors. RSC Adv..

[24] Suhas (2016). Cellulose: A review as natural, modified and activated carbon adsorbent. Biores. Technol..

[25] Yahya MA, Al-Qodah Z, Ngah CWZ (2015). Agricultural bio-waste materials as potential sustainable precursors used for activated carbon production: A review. Renew. Sustain. Energy Rev..

[26] Jain A, Balasubramanian R, Srinivasan MP (2016). Hydrothermal conversion of biomass waste to activated carbon with high porosity: A review. Chem. Eng. J..

[27] Ahammad AJS (2019). Activated jute carbon paste screen-printed FTO electrodes for nonenzymatic amperometric determination of nitrite. J. Electroanal. Chem..

[28] Deb Nath NC, Shah SS, Qasem MAA, Zahir MH, Aziz MA (2019). Defective Carbon Nanosheets Derived from *Syzygium cumini* Leaves for Electrochemical Energy-Storage. ChemistrySelect.

[29] Wang L (2014). Electrochemical Sensing and Biosensing Platform Based on Biomass-Derived Macroporous Carbon Materials. Anal. Chem..

[30] Hinks NJ, McKinlay AC, Xiao B, Wheatley PS, Morris RE (2010). Metal organic frameworks as NO delivery materials for biological applications. Microporous Mesoporous Mater..

[31] field survey and in vivo X-ray analyses (2011). Harada, E. *et al.* Assessment of willow (*Salix* sp.) as a woody heavy metal accumulator. Metallomics.

[32] Shah SS (2019). Preparation and characterization of manganese oxide nanoparticles-coated *Albizia procera* derived carbon for electrochemical water oxidation. J. Mater. Sci: Mater. Electron..

[33] Thurston EL (1974). Morphology, Fine Structure, and Ontogeny of the Stinging Emergence of *Urtica Dioica*. Am. J. Bot..

[34] Suman S, Panwar DS, Gautam S (2017). Surface morphology properties of biochars obtained from different biomass waste. Energy Sources, Part A: Recovery, Utilization, and Environmental Effects.

[35] Mutyala, S. & Jayaraman, M. Synthesis of Nitrogen Doped Carbon and Its Enhanced Electrochemical Activity towards Ascorbic Acid Electrooxidation. *Int. J. Electrochem.* https://www.hindawi.com/journals/ijelc/2014/246746/ (2014) 10.1155/2014/246746.

[36] Zhang ZH, Zhao TS, Bai BF, Zeng L, Wei L (2017). A highly active biomass-derived electrode for all vanadium redox flow batteries. Electrochim. Acta.

[37] Shang H (2015). Preparing high surface area porous carbon from biomass by carbonization in a molten salt medium. RSC Adv..

[38] Sodtipinta J (2017). Interconnected open-channel carbon nanosheets derived from pineapple leaf fiber as a sustainable active material for supercapacitors. Ind. Crops Prod..

[39] Chu M (2019). Novel biomass-derived smoke-like carbon as a supercapacitor electrode material. R. Soc. Open Sci..

[40] Rezma S, Birot M, Hafiane A, Deleuze H (2017). Physically activated microporous carbon from a new biomass source: Date palm petioles. C. R. Chim..

[41] Puech P (2019). Analyzing the Raman Spectra of Graphenic Carbon Materials from Kerogens to Nanotubes: What Type of Information Can Be Extracted from Defect Bands?. C Journal of Carbon Research.

[42] Gu W, Sevilla M, Magasinski A, Fuertes AB, Yushin G (2013). Sulfur-containing activated carbons with greatly reduced content of bottle neck pores for double-layer capacitors: a case study for pseudocapacitance detection. Energy Environ. Sci..

[43] Guerriero G, Sergeant K, Hausman J-F (2013). Integrated *-omics*: a powerful approach to understanding the heterogeneous lignification of fibre crops. Int J Mol Sci.

[44] Behr M (2019). Distribution of cell-wall polysaccharides and proteins during growth of the hemp hypocotyl. Planta.

[45] Thommes M (2015). Physisorption of gases, with special reference to the evaluation of surface area and pore size distribution (IUPAC Technical Report). Pure Appl. Chem..

[46] Ponomarev N, Sillanpää M (2019). Combined chemical-templated activation of hydrolytic lignin for producing porous carbon. Ind. Crops Prod..

[47] Sing, K. S. W. *et al.* Reporting Physisorption Data for Gas/Solid Systems With Special Reference to the Determination of Surface Area and Porosity.

[48] Morishita T (2010). A review of the control of pore structure in MgO-templated nanoporous carbons. Carbon.

[49] Nair R (2012). Effect of carbon nanomaterials on the germination and growth of rice plants. J Nanosci Nanotechnol.

[50] Jordan JT (2020). Carbon nanotubes affect early growth, flowering time and phytohormones in tomato. Chemosphere.

[51] López-Vargas ER (2020). Seed Priming with Carbon Nanomaterials to Modify the Germination, Growth, and Antioxidant Status of Tomato Seedlings. Agronomy.

[52] Tardieu F, Tuberosa R (2010). Dissection and modelling of abiotic stress tolerance in plants. Curr. Opin. Plant Biol..

[53] Bressan R, Bohnert H, Zhu J-K (2009). Abiotic stress tolerance: from gene discovery in model organisms to crop improvement. Mol Plant.

[54] Hedhly A, Hormaza JI, Herrero M (2009). Global warming and sexual plant reproduction. Trends Plant Sci..

[55] Mesihovic A, Iannacone R, Firon N, Fragkostefanakis S (2016). Heat stress regimes for the investigation of pollen thermotolerance in crop plants. Plant Reprod.

[56] Kroeger JH, Geitmann A (2012). Pollen tube growth: Getting a grip on cell biology through modeling. Mech. Res. Commun..

[57] Sabrine, H., Afif, H., Mohamed, B., Hamadi, B. & Maria, H. Effects of cadmium and copper on pollen germination and fruit set in pea (*Pisum sativum* L.). (2010).

[58] Wolters JHB, Martens MJM (1987). Effects of Air Pollutants on Pollen. Bot. Rev..

[59] Speranza A, Leopold K, Maier M, Taddei AR, Scoccianti V (2010). Pd-nanoparticles cause increased toxicity to kiwifruit pollen compared to soluble Pd(II). Environ. Pollut..

[60] Speranza A (2013). *In vitro* toxicity of silver nanoparticles to kiwifruit pollen exhibits peculiar traits beyond the cause of silver ion release. Environ Pollut.

[61] Hoecke KV, Schamphelaere KACD, der Meeren PV, Lcucas S, Janssen CR (2008). Ecotoxicity of silica nanoparticles to the green alga *Pseudokirchneriella subcapitata*: Importance of surface area. Environ. Toxicol. Chem..

[62] Katsumata M, Koike T, Nishikawa M, Kazumura K, Tsuchiya H (2006). Rapid ecotoxicological bioassay using delayed fluorescence in the green alga *Pseudokirchneriella subcapitata*. Water Res..

[63] Zhang L, Lei C, Yang K, White JC, Lin D (2018). Cellular response of *Chlorella pyrenoidosa* to oxidized multi-walled carbon nanotubes. Environ. Sci. Nano.

[64] Schwab F (2011). Are Carbon Nanotube Effects on Green Algae Caused by Shading and Agglomeration?. Environ. Sci. Technol..

[65] Saxena P (2020). Aquatic nanotoxicology: impact of carbon nanomaterials on algal flora. Energ. Ecol. Environ..

[66] Karakoti AS, Hench LL, Seal S (2006). The potential toxicity of nanomaterials—The role of surfaces. JOM.

[67] Fu PP, Xia Q, Hwang H-M, Ray PC, Yu H (2014). Mechanisms of nanotoxicity: Generation of reactive oxygen species. J. Food Drug Anal..

[68] Hull MS (2009). Release of Metal Impurities from Carbon Nanomaterials Influences Aquatic Toxicity. Environ. Sci. Technol..

[69] Pikula K (2020). Comparison of the Level and Mechanisms of Toxicity of Carbon Nanotubes, Carbon Nanofibers, and Silicon Nanotubes in Bioassay with Four Marine Microalgae. Nanomaterials.

[70] Bredemann, G. *Die grosse Brennessel Urtica dioica L.: Forschungen über ihren Anbau zur Fasergewinnung; mit einem Anhang über ihre Nutzung für Arznei- und Futtermittel sowie technische Zwecke von Kurt Garber*. (Akademie Verlag, 1959).

[71] Guerriero G (2017). Transcriptomic profiling of hemp bast fibres at different developmental stages. Sci. Rep..

[72] Blake AW (2006). Understanding the biological rationale for the diversity of cellulose-directed carbohydrate-binding modules in prokaryotic enzymes. J. Biol. Chem..

[73] Brewbaker JL, Kwack BH (1963). The Essential Role of Calcium Ion in Pollen Germination and Pollen Tube Growth. Am. J. Bot..

[74] OECD. Test No. 201: Freshwater Alga and Cyanobacteria, Growth Inhibition Test in OECD Guidelines for the Testing of Chemicals, Section 2, (OECD Publishing, Paris, 2011).

