# Supplementary material for: Physico-chemical properties and toxicological effects on plant and algal models of carbon nanosheets from a nettle fibre clone
Source: Sci Rep. 2021 Mar 25;11:6945. doi: 10.1038/s41598-021-86426-5 (PMC7994820; doi:10.1038/s41598-021-86426-5)
Supplement: Supplementary file 1 — Supplementary Information [file 41598_2021_86426_MOESM1_ESM.pdf]

Physico-chemical properties and toxicological effects on plant and algal models of carbon nanosheets from a nettle fibre clone

Syed Shaheen Shah[1], Mohammed Ameen Ahmed Qasem[1], Roberto Berni[2,3], Cecilia Del Casino[2], Giampiero Cai[2], Servane Contal[4], Irshad Ahmad[5], Khawar Sohail Siddiqui[6], Edoardo Gatti[7], Stefano Predieri[7], Jean-Francois Hausman[8], Sébastien Cambier[4], Gea Guerriero[8,*] and Md. Abdul Aziz[1,*]

[1]Center of Research Excellence in Nanotechnology, King Fahd University of Petroleum and Minerals, Dhahran 31261, Saudi Arabia.

[2]University of Siena, Department of Life Sciences, via P.A. Mattioli 4, I-53100 Siena, Italy.

[3]TERRA Teaching and Research Center, Gembloux Agro-Bio Tech, University of Liège, 5030 Gembloux, Belgium.

[4]Environmental Research and Innovation (ERIN) Department, Luxembourg Institute of Science and Technology (LIST), 5, avenue des Hauts-Fourneaux, Esch-sur-Alzette, Luxembourg.

[5]Life Sciences Department, King Fahd University of Petroleum and Minerals (KFUPM), Dhahran 31261, Saudi Arabia.

[6]School of Biotechnology and Biomolecular Sciences (BABS), The University of New South Wales, Sydney, NSW2052, Australia.

[7]Institute of Bioeconomy (IBE), National Research Council, Via P. Gobetti, 101-I, I-40129, Bologna, Italy.

[8]Environmental Research and Innovation (ERIN) Department, Luxembourg Institute of Science and Technology, 5, rue Bommel, Z.A.E. Robert Steichen, L-4940, Hautcharage, Luxembourg.

[*]Authors to whom correspondence should be addressed: gea.guerriero@list.lu; maziz@kfupm.edu.sa

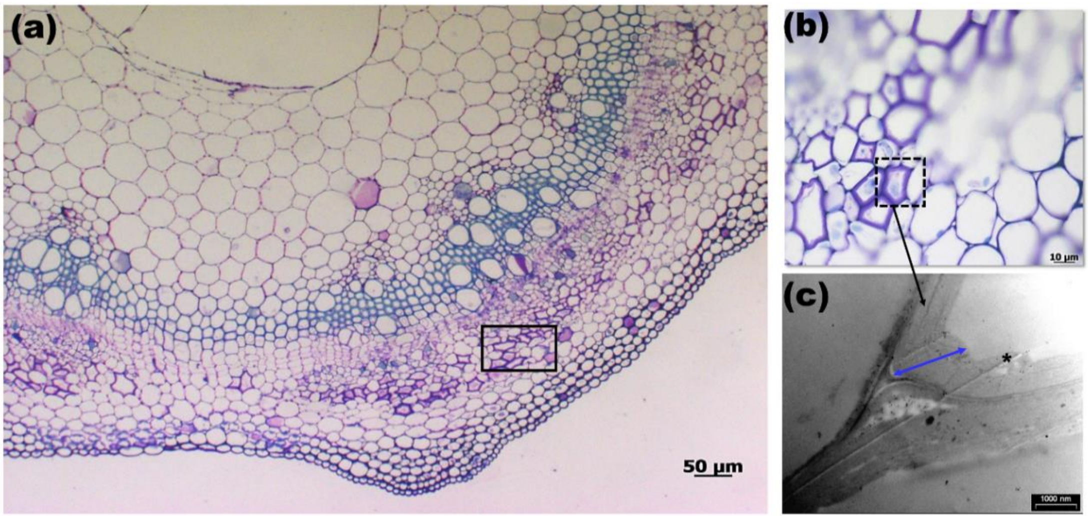

Supplementary Figure 1: Anatomy of the nettle stem. (a): Toluidine Blue O staining of a stem cross section (the square indicates bast fibres); (b): Magnified image showing a small cluster of three adjacent bast fibres (the dotted square indicates one bast fibre in transversal cross section); (c): immunoTEM image showing the gelatinous wall of nettle bast fibre (blue double-headed arrow) with gold nanoparticles denoting the presence of crystalline cellulose. The G-layer of nettle fibres shows a region with loose appearance (asterisk).

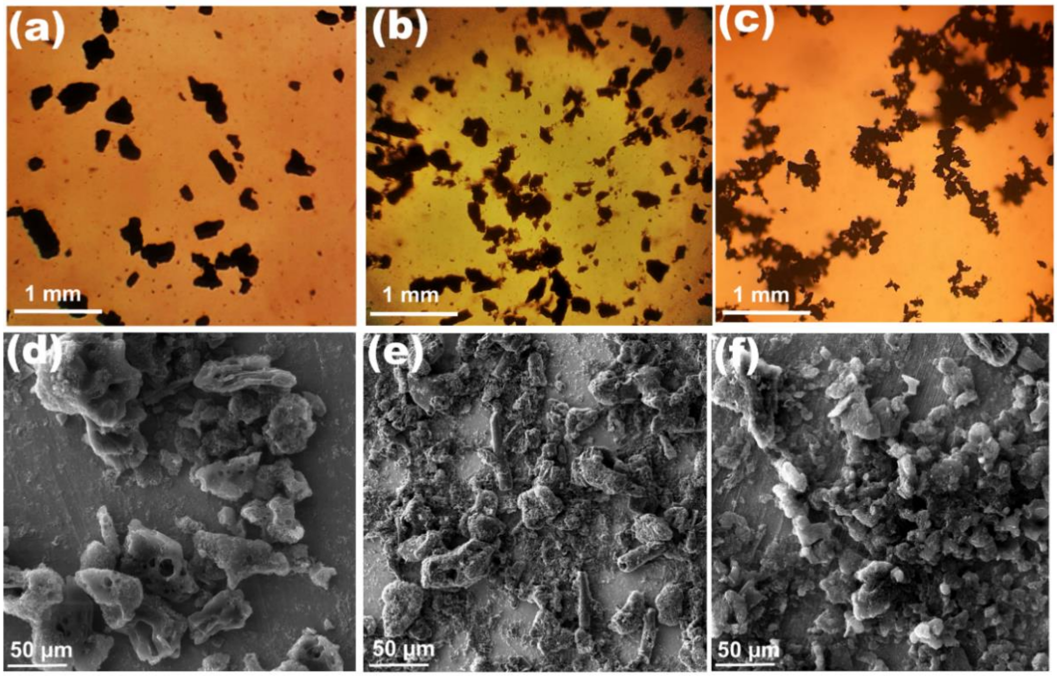

Supplementary Figure 2: Aggregates formed by the nettle CNS. (a) Stem-derived CNS, (b, c) leaf-derived CNS in BK_{suc} medium observed with an inverted microscope. FESEM images at low magnification of CNS prepared at 650 °C from nettle stem (d), leaves (e, f) and using $NaHCO_3$ as an activating agent. The ratio of nettle powder and $NaHCO_3$ is 1:1 (a, d and b, e) and 1:2 (c and f).

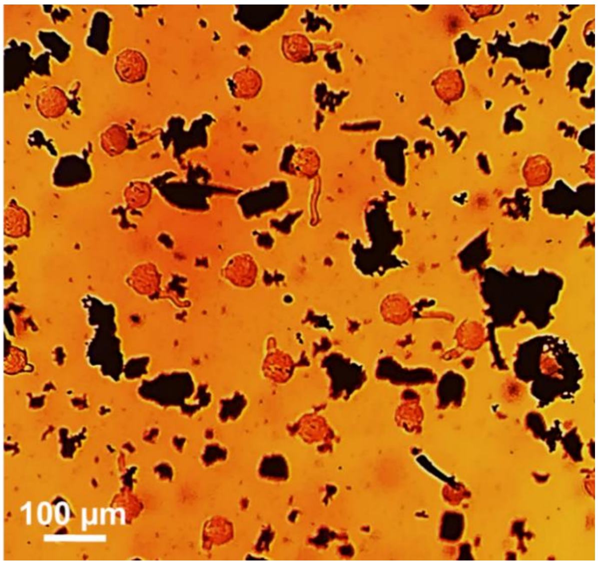

Supplementary Figure 3: Representative inverted microscopy image showing adhesion of CNS (in this case leaf CNS1:2 at 100 μg/mL) to the pollen grains.
